# Supplementary material for: Ligand-Assisted Formation of Graphene/Quantum Dot Monolayers with Improved Morphological and Electrical Properties
Source: Nanomaterials (Basel). 2020 Apr 11;10(4):723. doi: 10.3390/nano10040723 (PMC7221828; doi:10.3390/nano10040723)
Supplement: Supplementary file 1 [file nanomaterials-10-00723-s001.pdf]

Supplementary Materials

Ligand-Assisted Formation of Graphene/Quantum Dot Monolayers with Improved Morphological and Electrical Properties

Aleksandr P. Litvin [1,*], Anton A. Babaev [1], Peter S. Parfenov [1], Aliaksei Dubavik [1], Sergei A. Cherevkov [1], Mikhail A. Baranov [1], Kirill V. Bogdanov [1], Ivan A. Reznik [1], Pavel O. Ilin [1], Xiaoyu Zhang [2], Finn Purcell-Milton [3], Yurii K. Gun'ko [3], Anatoly V. Fedorov [1] and Alexander V. Baranov [1]

[1] Center of Information Optical Technology, ITMO University, 197101 St. Petersburg 197101, Russia; a.a.babaev@ifmo.ru (A.A.B.); psparfenov@itmo.ru (P.S.P.); adubavik@itmo.ru (A.D.); s.cherevkov@itmo.ru (S.A.C.); mbaranov@itmo.ru (M.A.B.); bogdanov.k@niuitmo.ru (K.V.B.); ivanreznik1993@itmo.ru (I.A.R.); 182845@niuitmo.ru (P.O.I.); a_v_fedorov@mail.ifmo.ru (A.V.F.); a_v_baranov@mail.ifmo.ru (A.V.B.)

[2] College of Materials Science and Engineering, Jilin University, Changchun 130012, China; zhangxiaoyu@jlu.edu.cn

[3] School of Chemistry and CRANN Trinity College Dublin, Dublin 2, Dublin D02 PN40, Ireland; fpurcell@tcd.ie (F.P.-M.); IGOUNKO@tcd.ie (Y.K.G.)

* Correspondence: litvin@itmo.ru

Received: 13 March 2020; Accepted: 8 April 2020; Published: 11 April 2020

Figure S1

Figure S1 (a) shows a typical π-A isotherm of the dependence of the surface pressure (π) for the PbS QDs film on its area (A). The shape of the isotherm corresponds to the literature data indicating the formation of a closed packed film when the isotherm reaches a plateau [1]. First, the PbS QDs film was deposited at the surface pressure of 15 mN/m (point 1). The AMF image of the obtained film is shown in figure S1 (b) and corresponding cross-section and histogram are shown in figure S1 (c,d). It is seen that the film consists of 1-2 monolayers of QDs with some uncovered areas. It demonstrates that the surface pressure was not high enough for formation of a continuous film. For the next deposition, the film was formed at the surface pressure of 16.5 mN/m that corresponds to the isotherm area marked as point 2. The AMF image and corresponding cross-section for the obtained film are shown in figure S1 (e,f). In this case, the formation of continuous 1-2 monolayered QD film occurred. Thus, for fabrication of rGO-PbS QDs film, we choose a surface tension located at the plateau of the isotherm.

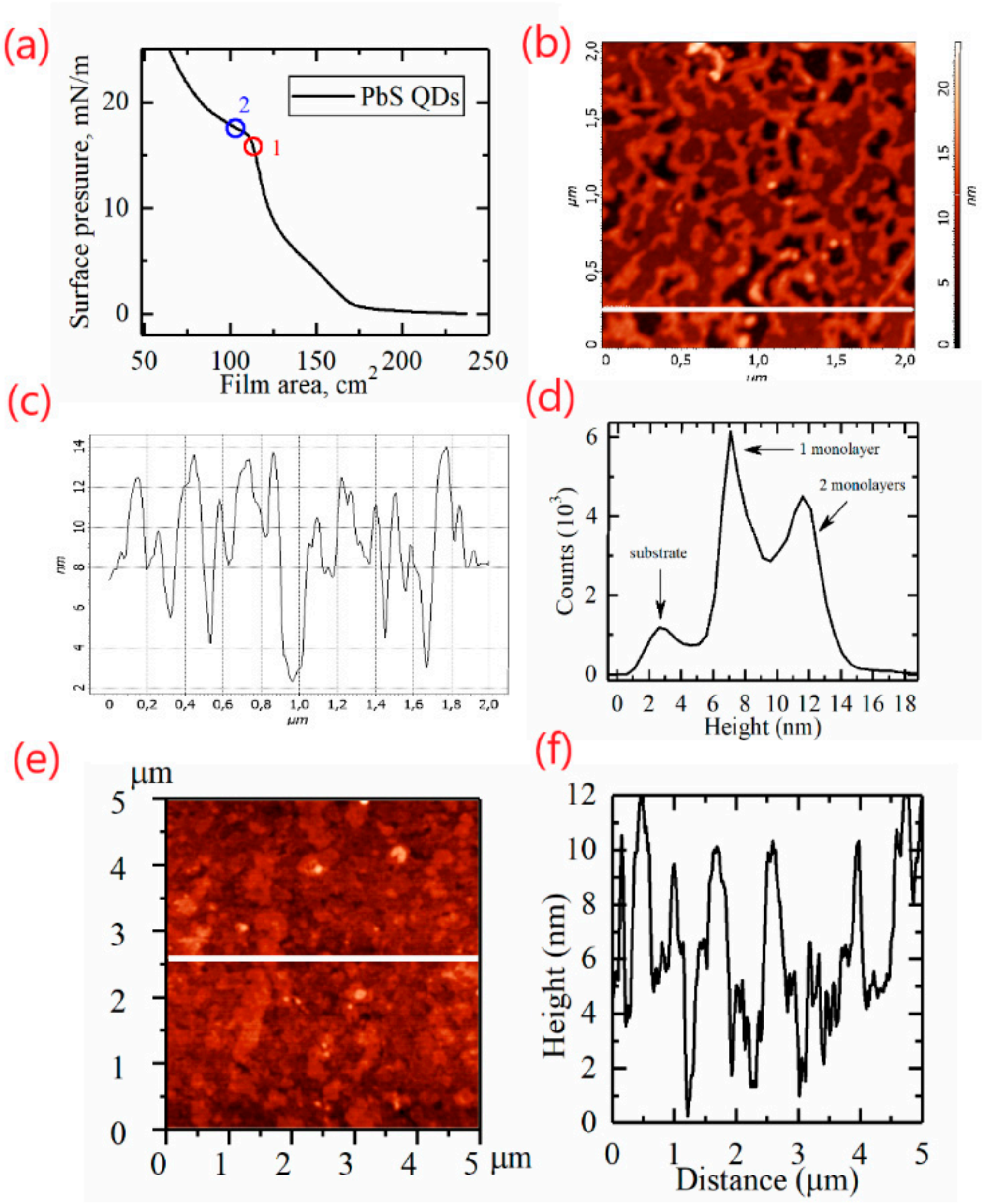

Figure S1-1. (**a**) a typical π-A isotherm of formation of the PbS QDs film; (**b–d**) the AMF image, corresponding cross-section and histogram for the film deposited at the surface tension of 15 mN/m (point 1); (**e,f**) the AMF image and corresponding cross-section for the film deposited at the surface tension of 16.5 mN/m (point 2).

Figure S1-2 shows the π-A isotherms of the dependence of the surface pressure (π) of the film on its area (A) that can be considered as typical for such materials in comparison with literature data [1]. Formation of the films made from the PbS(OA)-rGO and PbS(MPA)-rGO hybrid solutions was completed when the surface tension of the film reached 17 mN/m and 45 mN/m, respectively. The difference in the dynamics of the formation of the films of these materials is attributed to the different amount and composition of the ligand molecules leading to the formation of large aggregates in the case of MPA-capped PbS QDs.

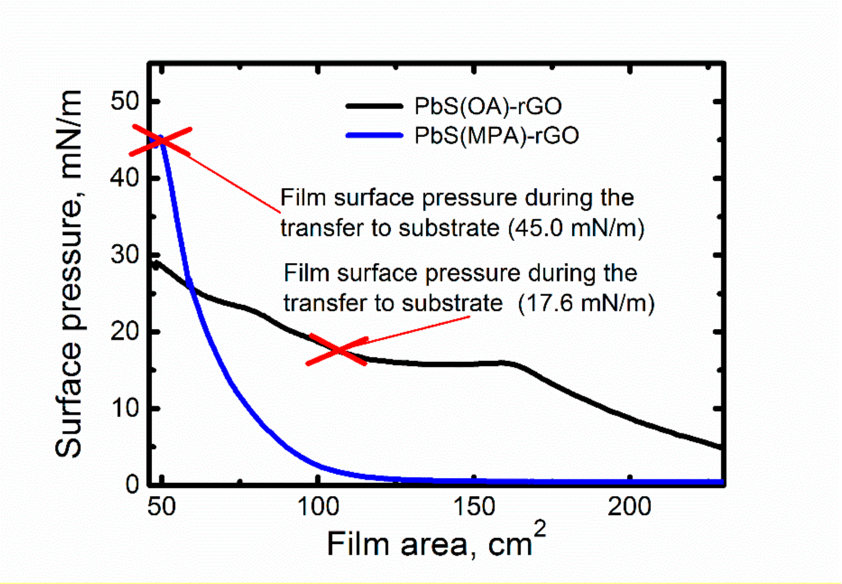

Figure S1-2. The π-A isotherms of formation of the films made from the PbS(OA)-rGO and PbS(MPA)-rGO hybrid solutions.

Figure S2

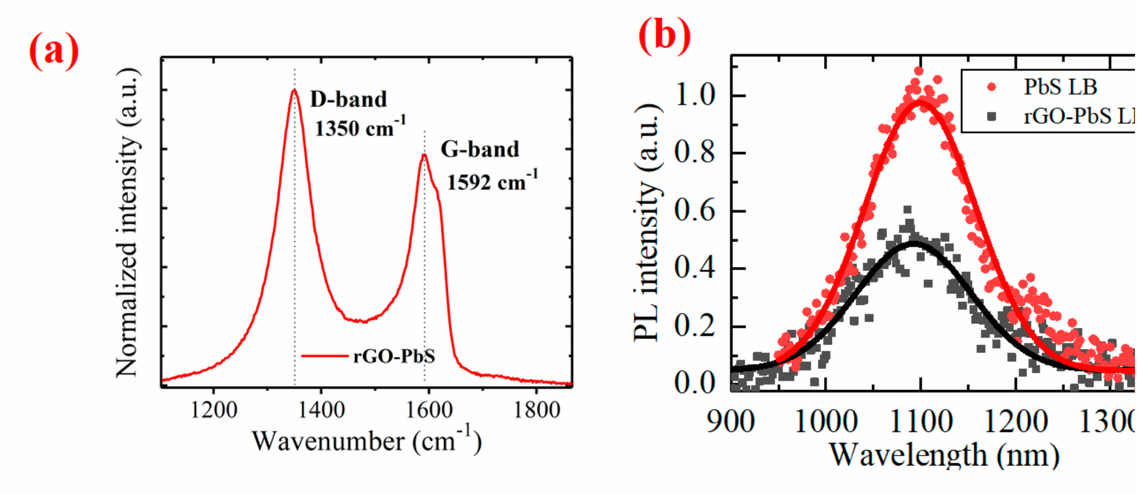

Figure S2. (**a**) the Raman spectra taken from a PbS-rGO LB film; (**b**) the PL spectra from pristine and hybrid LB films with 4.0 nm PbS QDs.

Figure S2 (a) shows the Raman spectra taken from a PbS-rGO LB film and measured using Renishaw micro-Raman spectrometer with the 457.9 line of the Ar+ laser as the excitation sources. The peaks centered at 1350 and 1592 cm^{-1} are related to the D and G bands of rGO, respectively, that is in a good agreement with a literature data [2,3]. Figure S2 (b) shows a typical PL spectrum from 4.0 nm PbS QDs in a hybrid LB film (black line) as compared to the PL spectrum from pristine QDs prepared by the LB method. The spectrum can be fitted by a Gaussian function. A slight blue shift of PL peak position is observed for both pristine and hybrid films as compared to the QD in solution. PL peak position is located at 1110±6 nm, 1100±9 nm, and 1092±9 nm for CCl_4 solution, pristine and hybrid films, respectively. A slight blue shift of PL peak position can be ascribed to fast oxidation of a thin QD layer [4,5]. For all the spectra, FWHM was kept at the value of 140-145 nm, indicating no water-induced formation of additional trap states.

Figure S3

PbS QDs capped with OA were treated with MPTS as a reference sample for estimation of charge transfer efficiency. PL decay for spin-coated PbS QDs treated with MPTS shown in a figure S3 by red circles can be well fitted by two exponential function with an average decay time of 0.61 μs. In a LB film, QDs demonstrate faster decay with an average decay time of 0.12 μs. An efficiency of charge transfer can be estimated as follows:

$$E_{ch\_tr} = 1 - \frac{\tau_{MPTS-rGO}}{\tau_{MPTS}},$$

where τ_{MPTS} and $\tau_{MPTS-rGO}$ are average PL lifetimes for the QDs treated with MPTS and QDs in a LB film. From the calculated PL lifetimes charge transfer efficiency as high as 80% can be estimated.

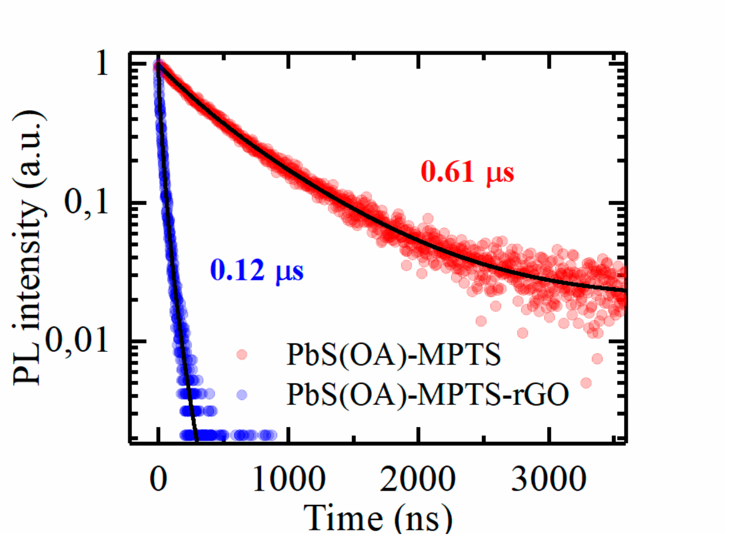

Figure S3. PL decay for PbS QDs capped with OA and treated with MPTS (red circles) and in a LB film (blue circles). Black lines show the fitting.

Figure S4

An average roughness of the films obtained by spin-coating and LB methods was estimated using AFM. Figure S4 shows 20 × 20 μm areas of the spin-coated (a) and LB (b) rGO-PbS QDs films. An average roughness was estimated to be 2.9 ± 1.0 nm and 1.7 ± 0.5 nm, respectively.

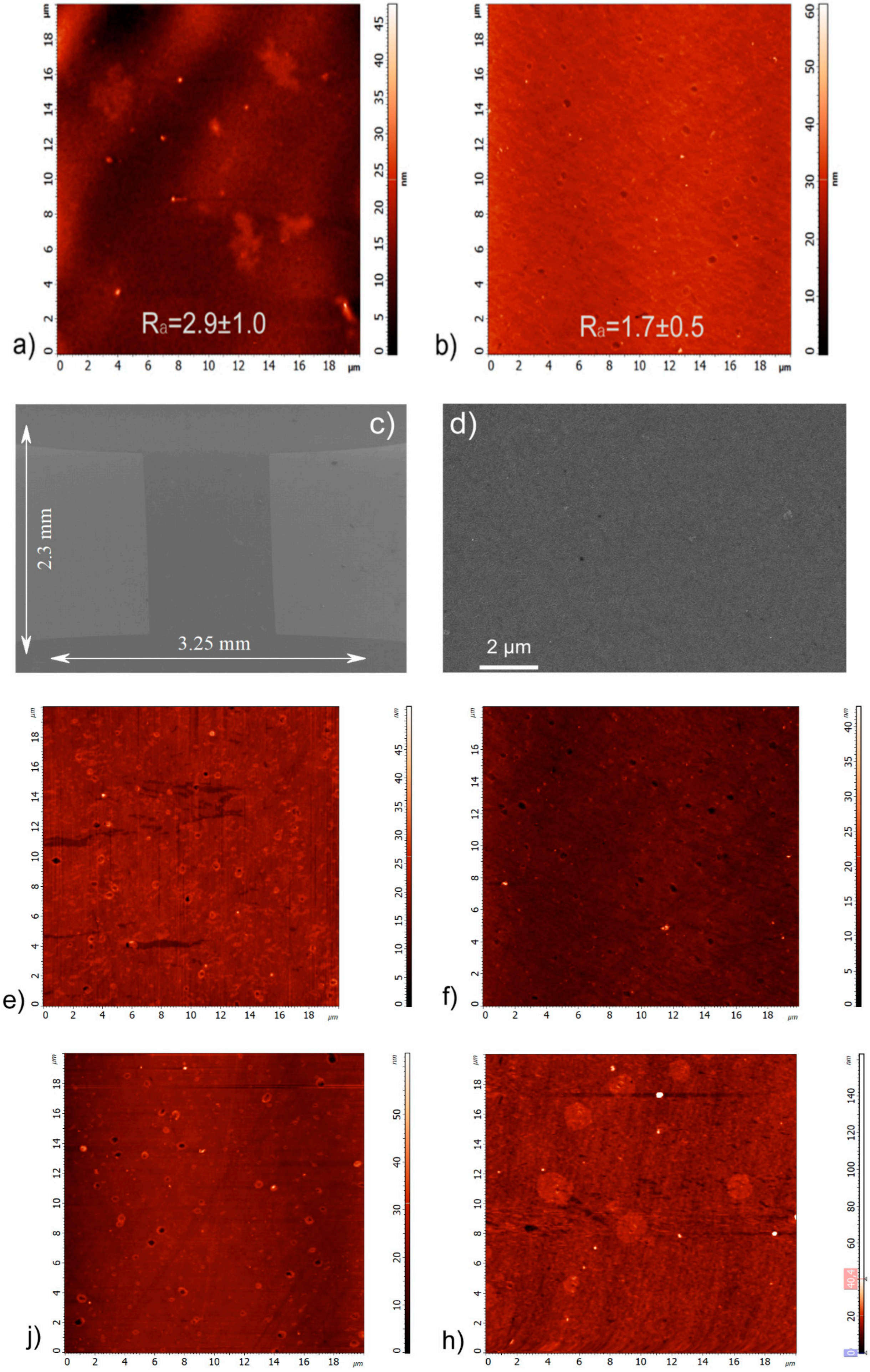
a)
Ra=2.9±1.0
b)
Ra=1.7±0.5
c)
2.3 mm
3.25 mm
d)
2 µm
e)
f)
j)
h)

Figure S4. AFM images of the spin-coated (**a**) and LB (**b**) rGO-PbS QDs films demonstrate better average roughness of the LB samples; (**c-d**) SEM images with different magnification obtained for the LB film deposited on patterned ITO substrate; (**e-j**) AFM images LB rGO-PbS QDs films taken from four random 20 × 20 μm² areas.

Figure S5.

A similar LB film formation procedure was used for PbS QDs with a previously replaced ligand shell. First, MAI and MPA were used for a colloidal-phase ligand exchange. Then, QDs were attached to rGO with MPTS. After film fabrication, very pure quality of the films was observed by SEM (figure S4 (a,b)). To check the influence of oleic acid on film formation, the reduced PbS/rGO ratio was used. The obtained island-like film is shown in a figure S4(c).

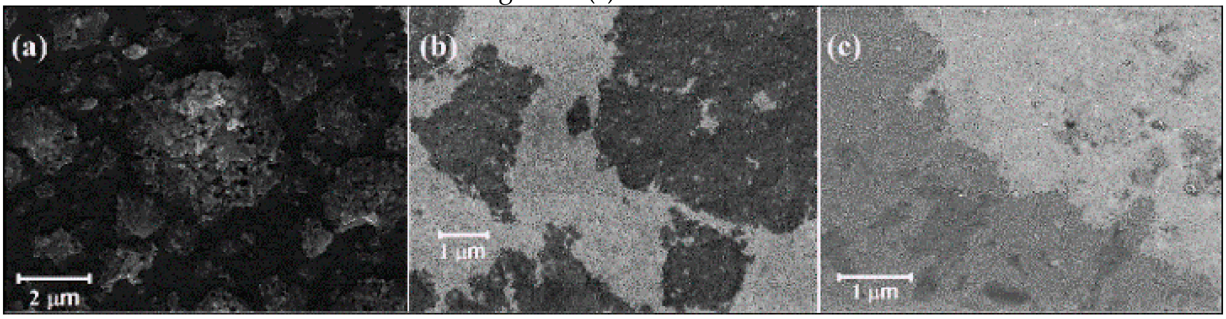

Figure S5. SEM images of LB films prepared from the hybrid solutions of MPTS rGO with MAI-PbS (**a**), MPA-PbS (**b**), and OA-PbS (using a reduced ratio of 1/15 rGO/PbS ratio, **c**).

Figure S6.

After the post-deposition ligand-exchange, SEM images were taken to check the quality of film surface. It was found, that both EDT and TBAI ligand-exchange processes do not worsen the film quality, as shown in figure S4. No additional cracks or pinholes was found after the ligand-exchange procedures.

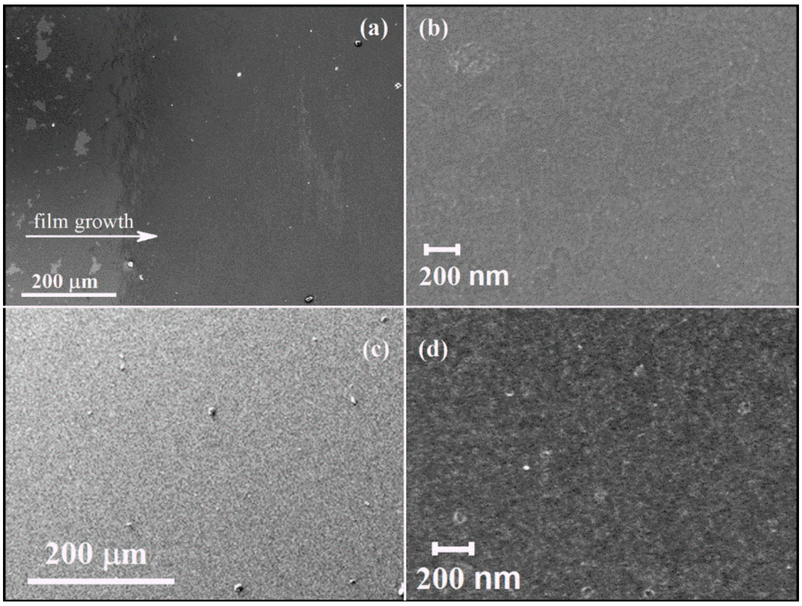

Figure S6. SEM images of rGO-PbS LB films treated with EDT (a,b) and TBAI (c,d).

Figure S7.

The conductivity under light illumination was measured for the LB films treated with EDT. The curves were compared to those obtained for OA-capped QDs deposited by spin-coating. It can be seen that the LB films demonstrate an increase of current under white light illumination.

At the bias of 5 V, the EDT-PbS showed the responsivity of 2.8×10^{-6} A/W with the dark current of 0.53 nA and the TBAI-PbS showed a responsivity of 2.6×10^{-6} A/W with a dark current of 0.29 nA. According to the literature data, the responsivity of graphene-based photodetectors spans the range from 10^{-6} A/W (rGO, GO) to 10^{8} A/W (CVD-grown graphene) [6,7]. The low obtained value of responsivity can be attributed to several factors. First, rGO- and GO-based photodetectors normally demonstrate lower values of responsivity [6]. Secondly, an extremely thin layer of QDs was used. Usually, graphene-PbS photodetectors have an absorbing layer thicker than 100 nm [8]. Thirdly, the calculated responsivity characterizes an active area of 24 mm^2 which consists of numerous coupled rGO flakes. In contrast to that, much higher responsivity can be achieved when the device is fabricated in a single graphene flake with a size of tens of μm [9].

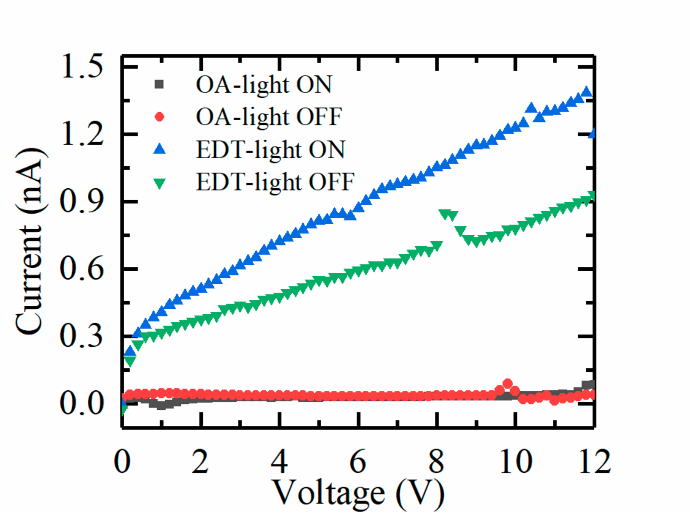

Figure S7. IV-curves obtained for the EDT-treated LB films as compared to OA-capped QDs deposited by spin-coating.

References

1. Justo, Y.; Moreels, I.; Lambert, K.; Hens, Z. Langmuir-Blodgett monolayers of colloidal lead chalcogenide quantum dots: morphology and photoluminescence. *Nanotech.* **2010**, *21*, 295606.

2. Hidayah, N.M.S.; Liu, W.W.; Lai, C.W.; Noriman, N.Z.; Khe, C.S.; Hashim, U.; Lee, H.C. Comparison on graphite, graphene oxide and reduced graphene oxide: Synthesis and characterization. *AIP Conf. Proc.* **2017**, *1892*.

3. Martín-García, B.; Polovitsyn, A.; Prato, M.; Moreels, I. Efficient charge transfer in solution-processed PbS quantum dot-reduced graphene oxide hybrid materials. *J. Mater. Chem. C* **2015**, *3*, 7088–7095.

4. Litvin, A.P.; Parfenov, P.S.; Ushakova, E.V.; Fedorov, A.V.; Artemyev, M.V.; Prudnikau, A.V.; Golubkov, V.V.; Baranov, A.V. PbS quantum dots in a porous matrix: Optical characterization. *J. Phys. Chem. C* **2013**, *117*.

5. Skurlov, I.D.; Korzhenevskii, I.G.; Mudrak, A.S.; Dubavik, A.; Cherevkov, S.A.; Parfenov, P.S.; Zhang, X.; Fedorov, A. V.; Litvin, A.P.; Baranov, A. V. Optical properties, morphology, and stability of iodide-passivated lead sulfide quantum dots. *Materials (Basel).* **2019**, *12*.

6. De Sanctis, A.; Mehew, J.D.; Craciun, M.F.; Russo, S. Graphene-based light sensing: Fabrication, characterisation, physical properties and performance. *Materials (Basel).* 2018, *11*.

7. Wu, J.; Lu, Y.; Feng, S.; Wu, Z.; Lin, S.; Hao, Z.; Yao, T.; Li, X.; Zhu, H.; Lin, S. The Interaction between

Quantum Dots and Graphene: The Applications in Graphene-Based Solar Cells and Photodetectors. *Adv. Funct. Mater.* **2018**, *28*, 1804712.

8. Che, Y.; Zhang, Y.; Cao, X.; Song, X.; Zhang, H.; Cao, M.; Dai, H.; Yang, J.; Zhang, G.; Yao, J. High-performance PbS quantum dot vertical field-effect phototransistor using graphene as a transparent electrode. *Appl. Phys. Lett.* **2016**, *109*, 263101.

9. Konstantatos, G.; Badioli, M.; Gaudreau, L.; Osmond, J.; Bernechea, M.; Garcia de Arquer, F.P.; Gatti, F.; Koppens, F.H.L. Hybrid graphene-quantum dot phototransistors with ultrahigh gain. *Nat. Nanotechnol.* **2012**, *7*, 363–8.
